# Metabolically Healthy Overweight and Obesity Is Associated with Higher Adherence to a Traditional Dietary Pattern: A Cross-Sectional Study among Adults in Lebanon

**DOI:** 10.3390/nu8070432

**Published:** 2016-07-20

**Authors:** Joane Matta, Lara Nasreddine, Lamis Jomaa, Nahla Hwalla, Abla Mehio Sibai, Sebastien Czernichow, Leila Itani, Farah Naja

**Affiliations:** 1 Department of Nutrition, Faculty of Agricultural and Food Sciences, Holy Spirit University, P.O. Box 446, Jounieh, Lebanon; joanematta@usek.edu.lb; 2 Department of Nutrition and Food Science, Faculty of Agricultural and Food Sciences and Nutrition for Health Program (NHP), Office of Strategic Health Initiatives American University of Beirut, Beirut 1107-2020, Lebanon; ln10@aub.edu.lb (L.N.); lj18@aub.edu.lb (L.J.); Nahla@aub.edu.lb (N.H.); 3 Department of Epidemiology and Population Health, American University of Beirut, Beirut 1107-2020, Lebanon; am00@aub.edu.lb; 4 INSERM, UMS 011, Villejuif UMS 011, France; sebastien.czernichow@aphp.fr; 5 Department of Nutrition, Hopital Europeen Georges Pompidou, Paris 75015, France; 6 School of Medicine, Paris Descartes University, Paris 75006, France; 7 Department of Nutrition & Dietetics, Faculty of Health Sciences, Beirut Arab University, P.O. Box 11-5020 Riad El Solh, Beirut 11072809, Lebanon; itani.leila@gmail.com

**Keywords:** metabolically healthy obesity, Lebanese dietary pattern, traditional dietary pattern

## Abstract

This study aimed to examine the proportion and socio-demographic correlates of Metabolically Healthy Overweight and Obesity (MHOv/O) among Lebanese adults and to investigate the independent effect of previously identified dietary patterns on odds of MHOv/O. Data were drawn from the National Nutrition and Non-Communicable Disease Risk Factor Survey (Lebanon 2008–2009). Out of the 337 adult participants who had complete socio-demographic, lifestyle, dietary as well as anthropometric and biochemical data, 196 had a BMI ≥ 25 kg/m^2^ and their data were included in this study. MHOv/O was identified using the Adult Treatment Panel criteria. Dietary patterns previously derived in this study population were: Fast Food/Dessert, Traditional-Lebanese and High-Protein. The proportion of MHOv/O in the study sample was 37.2%. Females, higher education and high level of physical activity were positively associated with odds of MHOv/O. Subjects with higher adherence to the Traditional-Lebanese pattern had higher odds of MHOv/O (OR: 1.83, 95% CI: 1.09–3.91). No significant associations were observed between the Fast Food/Dessert and the high-protein patterns with MHOv/O. Follow-up studies are needed to confirm those findings and understand the mechanisms by which the Traditional-Lebanese pattern may exert a protective effect in this subgroup of overweight and obese adults.

## 1. Introduction

Obesity is generally associated with numerous metabolic disorders such as insulin resistance (IR) and type 2 diabetes [1], hypertension [2,3,4], dyslipidemia, and some forms of cancer [5,6,7]. However, studies have shown that a subset of overweight and obese individuals does not display such cardio-metabolic abnormalities and were identified under a phenotype termed ‘Metabolically Healthy Obese: MHO’. Those individuals present with favorable metabolic profiles such as insulin sensitivity, normal blood pressure and lipids profiles [8]. Additional protective factors identified among individuals with MHO include lower levels of circulating C-reactive protein (CRP) [9] and a possible elevation of adiponectin [10]. It has been suggested that this subset of overweight and obese subjects may also have a lower risk of mortality and a better prognosis than their non-metabolically healthy obese counterparts [11,12]. 

The prevalence of MHO varies according to the definitions used to characterize this phenotype and could reach up to 40% among obese adults [13,14]. Given the possible protective effects of MHO on morbidity and its considerable prevalence, the identification of underlying characteristics that may be associated with it may help provide a better understanding of the factors that may protect overweight and obese individuals from developing metabolic disturbances [12,15]. Some studies have shown that the prevalence of MHO is higher among younger vs. older individuals and among women vs. men [16,17]. Physical activity has been suggested as one of the lifestyle factors that may be associated with a metabolically healthy profile among overweight and obese subjects [16,17]. Similarly, it was suggested that specific dietary factors may be associated with the MHO phenotype, even though the number of studies in this field is still limited [18]. For instance, a study conducted among adult overweight and obese women showed that the MHO phenotype was positively associated with higher daily intakes of fiber and a higher number of daily servings of vegetables [18]. However, subsequent studies failed to confirm similar associations [19,20]. Results from a recent investigation of food groups, macro- and micronutrients’ intakes in a multi-ethnic group of 775 obese Americans did not support the hypothesis that dietary intake is associated with MHO [21]. This controversy may partly result from the use of traditional methods in nutritional epidemiology which focuses on the intake of individual nutrients, foods or food groups when investigating the association between diet and MHO. Such a conventional approach has several limitations mainly the interaction between nutrients, confounding by foods/nutrients not eaten and the problem of collinearity [22]. In this context, and to overcome the limitations of the traditional methods of examining single foods or nutrients, dietary pattern analysis was proposed as an approach that allows the examination of the holistic effect of diet on disease [23]. In fact, the dietary pattern approach looks beyond single nutrients or foods and attempts to capture the broader picture of diet that is hypothesized to be linked to health. In addition, results stemming from dietary patterns analyses are more helpful in disseminating diet-related messages to consumers who seem more likely to adhere to these messages rather than those stemming related to single foods or nutrients [22]. 

In Lebanon, similarly to other Eastern Mediterranean countries, the rate of overweight and obesity among adults is reported to follow an escalating secular trend, with its prevalence estimates increasing from 54.4% to 65.0% over the past decade [24]. Such an alarming trend coupled with the potential protective effect of MHO on disease risk, underscores the need to examine the proportions of overweight and obese individuals who are metabolically healthy and to investigate associated dietary and lifestyle factors. This study aims to (1) examine the proportion of Metabolically Healthy Overweight and Obesity (MHOv/O) among Lebanese adults (2) evaluate the socio-demographic and lifestyle correlates of MHOv/O; and (3) investigate the independent effect of previously identified dietary patterns on odds of MHOv/O in the study population. 

## 2. Materials and Methods 

### 2.1. Study Design and Participants

Data for this study were drawn from the cross-sectional National Nutrition and Non-Communicable Disease Risk Factor Survey (2008–2009) described elsewhere [25]. In brief, households, the primary sampling units, were drawn using a stratified cluster random sampling frame. The strata were the six administrative Lebanese governorates, while the clusters were further selected at the level of districts. Using the household roster, one adult from each household was selected. The distribution of the study sample by sex and 5-year age group was similar to that of the Lebanese population as estimated by the Central Administration for Statistics in Lebanon (2004) [26]. Out of 2202 visited households, 1982 accepted to participate in the study (response rate 90%) [27]. Of those, participants who had no chronic diseases and were not taking blood pressure, sugar or lipid lowering medications were contacted to give blood samples (*n* = 1331). From these participants, 337 subjects provided written consent and gave a blood sample. Comparison of subjects who gave blood and those who did not showed that both groups were comparable across the sociodemographic characteristics except for marital status (62% of respondents vs. 50% of non-respondents are married). In addition, significantly higher proportions of overweight and obesity were found among those who gave blood as compared to those who did not (Overweight 36%, Obesity: 22% vs. Overweight 33%, Obesity 16% respectively, *p* < 0.05). (Unpublished data). Of these 337 subjects, 196 were overweight and obese individuals (BMI ≥ 25 kg/m^2^), and their data are included in this study (overweight: *n* = 119; obese: *n* = 77). 

### 2.2. Data Collection

At the participants’ homes, data collection was conducted by trained field workers, phlebotomists and dietitians. Data collection procedures followed the WHO STEP wise approach to Surveillance (STEPS) [26] and included socio-demographic and lifestyle questionnaires, anthropometric measurements, biochemical assessment, as well as a food frequency questionnaire (FFQ) for the evaluation of dietary intake. The study protocol was reviewed and approved by the Institutional Review Board of the American University of Beirut, and informed consent was obtained from all participants in the study. 

Socio-demographic characteristics were age, sex, marital status, education level, crowding index, and family history of obesity. Crowding index was defined as the average number of people per room, excluding the kitchen and bathroom. Previous studies have shown that a higher crowding index was correlated with a lower socioeconomic status [28,29,30]. This finding was further validated in the Lebanese context [31]. A positive family history of obesity was defined as either one of the two parents (mother or father) identified as obese. Lifestyle factors included smoking, physical activity, weekly frequency of breakfast, snack consumption as well as frequency of eating at TV and eating out. Snacking was defined as an intake occasion that was not considered a main meal, light meal/breakfast or drink-only [32]. ‘Eating out’ and ‘Eating at TV’ were examined in terms of number of occasions per week. Physical activity was assessed using the short version of the International Physical Activity Questionnaire, and three levels of physical activity were determined based on metabolic equivalents-min per week (low, moderate, high) [33]. 

Anthropometric measurements obtained included weight, height and waist circumference, all of which were obtained using standardized protocols. The percent body fat was computed using skinfold thickness measurements according to the Durnin and Womersley formula [34]. Blood pressure was measured using a standard mercury sphygmomanometer. Two readings were obtained for both systolic and diastolic blood pressure, at 5-min intervals, and their average was used in this study. 

Metabolic and biochemical assessments were measured after an overnight fast and included serum triglycerides, HDL and LDL-cholesterol, fasting blood glucose, fasting blood insulin, and CRP. Details regarding blood collection and analysis were described elsewhere [25]. 

Dietary patterns in the study population: Previous work by our research group derived dietary patterns among survey participants who had complete socio-demographic, lifestyle, dietary as well as anthropometric and biochemical data [25]. The FFQ used to assess dietary intake had 61 items and measured food intake over the past year. For each food item listed in the FFQ, a standard portion size was indicated and five frequency choices were given. This FFQ was designed by a panel of nutritionists and included culture specific dishes and recipes. It was tested on a convenient sample to check for clarity and cultural sensitivity. For the derivation of the dietary patterns, food items were grouped into 25 food groups based on similarities in ingredients, nutrient profile, and/or culinary usage [25]. Food items having a unique composition (e.g., eggs, olives, and mayonnaise) were classified individually. The total consumption for each group was determined by summing the daily gram intake of each item within the group. Using the dietary intake of these 25 food groups, dietary patterns were identified by factor analysis. The latter is a data-driven technique which identifies foods that are frequently consumed together by grouping food items based on the degree to which the amounts eaten are correlated together [35]. The number of factors/patterns to be retained was based on three criteria: (1) the Kaiser criterion (eigenvalues > 1); (2) inflection point of the scree plot; and (3) the interpretability of factors. The rotated factor loadings matrix was extracted (Varimax rotation). The derived dietary patterns were labeled based on food groups having a rotated factor loading greater than 0.4. Factor scores were calculated by multiple regressions; a higher factor score indicated a greater adherence to the respective factor or pattern [36].

Accordingly, three dietary patterns were identified: Fast Food/Dessert, Traditional-Lebanese, and High Protein. The Fast Food/Dessert pattern consisted mainly of hamburger, shawarma, pizza and pies, falafel sandwiches, desserts, as well as carbonated beverages and juices. The Traditional-Lebanese pattern was identified as a variant of the Mediterranean diet [37] and included foods such as dairy products, olives, fruits, legumes, grains, eggs, vegetable oil, dried fruits, and traditional sweets. The High Protein pattern was characterized by high intakes of fish, chicken, meat, and low fat dairy products. Further details about the patterns and the loadings of each food items are presented in Appendix. The patterns’ scores for Fast Food/Dessert, Traditional-Lebanese, and High Protein of overweight and obese participants (BMI ≥ 25kg/m^2^) were included in the analyses of this study.

### 2.3. MHOv/O Classification

To date, there is no consensus on the MHO phenotype’s definition. A commonly used definition is The Adult Treatment Panel criteria for the metabolic syndrome (ATP-III) criterion, which was used in this study. Accordingly, an overweight or obese subject was classified as MHOv/O if he/she had one or none of the following: triglycerides ≥1.7 mmol/L; systolic blood pressure (BP) ≥130 mm·Hg; diastolic blood pressure ≥85 mm·Hg; fasting blood glucose ≥5.6 mmol/L; and HDL- cholesterol <1.04 mmol/L for men and <1.29 mmol/L for women. Overweight and obese subjects with more than one of these conditions were classified as Metabolically Unhealthy Overweight and Obese (MUHOv/O).

### 2.4. Statistical Analyses

Descriptive statistics for socio-demographic, lifestyle, anthropometric, and biochemical characteristics of study participants were presented as means ± SD and proportions for continuous and categorical variables, respectively. Chi-square and independent t-tests were used to compare MHO and MUHOv/O groups. The association of each of the characteristics of study participants with MHOv/O was assessed using simple logistic regression analysis. In order to evaluate the determinants of MHOv/O, a multiple logistic regression model was used. In this model, variables were included if they were significantly associated with the outcome in the univariate analysis. Simple and multiple logistic regression models were also used to evaluate the associations between the dietary patterns of the study population and the odds of the MHOv/O phenotype. In these models, scores of the dietary pattern were the independent variable (grouped as low adherence-1st tertile- and high adherence-2nd and 3rd tertile), and MHOv/O phenotype as dependent variable (MHOv/O vs. MUHOv/O). These models were adjusted for variables found to be significantly associated with MHOv/O. Tests for linearity (Tolerance > 0.4) of the covariates included in the regression models were performed. Normality of the residuals was assessed by the histogram of standardized residuals and normal probability plot in all regression models. All analyses were undertaken using SPPS software version 22 (IBM Corp. Released 2013. IBM SPSS Statistics for Windows, Version 22.0. IBM Corp: Armonk, NY, USA).

## 3. Results

The proportion of MHOv/O in the study population was 37.24%, 95% CI (30.78–44.19). Descriptive characteristics of the study population and their association with MHOv/O, as derived by simple logistic regression, are shown in Table 1. Of socio-demographic characteristics, sex, and education level were associated with MHOv/O status. Females and participants with a higher education level had higher odds of MHOv/O. Belonging to the ‘High Physical Activity’ category was also associated with a higher odd of MHOv/O. While BMI and percent body fat were not associated with MHOv/O, a one unit increase in waist circumference led to significantly lower odds of MHOv/O in the study population.

Correlates of the MHOv/O status were examined by multiple logistic regression, and the resulting OR and their corresponding 95% CI are presented in Table 2. After adjustment, female sex, higher education, and physical activity levels were associated with a higher odd of MHOv/O among study participants.

The OR and 95% CI for the association of adherence to these dietary patterns with the odds of MHOv/O are presented in Table 3. These odds were derived from multiple logistic regression adjusted for sex, education, and physical activity. Results indicated that a high adherence to the Traditional-Lebanese pattern was associated with 83% increase in the odds of MHOv/O (OR: 1.83, 95% CI: 1.09–3.91). No significant association was noted between the Fast Food/Dessert and the High Protein patterns and the odds of MHOv/O in the study population.

## 4. Discussion

This study is the first in Lebanon and the region to assess the proportion and correlates of MHOv/O and investigate its association with dietary patterns among overweight and obese adults. The results showed that the proportion of MHOv/O in the current study sample is 38.2%, implying that approximately one out of three overweight/obese individuals is considered metabolically healthy. This proportion is higher than a recent estimate from a prospective cohort of 4397 adults in Spain, where prevalence of MHOv/O was found to be 28.7% [38]. Such a difference could be attributed to the fact that overweight and obese participants with medical complications were not included in the denominator for calculating the proportion of MHOv/O in the study sample, given that subjects were excluded if they had a known history of chronic diseases and/or were taking blood pressure, sugar, or lipid lowering medications. Such an exclusion criterion was chosen to avoid reverse causality in the association between MHOv/O and dietary patterns.

The findings of this study showed that, even though BMI and percent body fat were not associated with MHOv/O, a negative association between waist circumference and MHOv/O was observed. Recognizing waist circumference as a proxy measure of visceral adiposity [39], these findings implicate a possible role for visceral fat in modulating the metabolic profile in overweight and obese subjects. In fact, it has been proposed that metabolically healthy individuals have lower amounts of visceral fats [40,41]. Abdominal obesity, with its characteristic increase in visceral fat, is associated with an increase in the levels of free fatty acids (FFA) and abnormal adipokine profiles, which can result in the development of insulin resistance and other metabolic abnormalities including dyslipidemia, hyperglycemia, and elevated blood pressure. 

In this study, investigation of the correlates of MHOv/O showed that female gender, higher education levels, and higher physical activity are significantly associated with higher odds of this phenotype. The fact that MHOv/O status was positively associated with female gender is in line with what has been reported in other investigations on MHO [40] and may be a reflection of the lower visceral fat deposition in women compared to men, when matched for BMI [42,43]. The positive association between female gender and MHOv/O is also in agreement with studies reporting women as being more health-conscious and followers of dietary recommendations than men [44,45]. Higher education may also be a driving factor towards healthier diets and lifestyles, and therefore higher odds of being metabolically healthy [46]. Our findings regarding the positive association between physical activity and MHOv/O are in accordance with what has been previously reported [13] and may be explained by several mechanisms including stimulation of fatty acid uptake and oxidation and increasing insulin sensitivity [47]. 

In the present study, we have opted to use the dietary pattern approach in investigating the association between diet and the MHOv/O phenotype. Previous studies conducted by our group on a national sample of Lebanese adults, which included normal weight, overweight and obese subjects, documented a positive association between metabolic syndrome and adherence to the Fast Food/Dessert pattern, a pattern that contains most of the food groups characteristic of the “Western Pattern” [25,48] and which was found to be associated with higher intakes of fat, saturated fat, sugar, and sodium coupled with lower intakes of dietary fiber and calcium, when compared to traditional Lebanese dietary pattern [25]. However, in the present study, which focuses on overweight and obese subjects only, we did not observe an association between the MHOv/O status and adherence to the Fast Food/Desert pattern. These results suggest that the negative effects of the Fast Food/Desert pattern on cardio-metabolic risk and the metabolic syndrome could be mediated mainly by its effects on increasing adiposity and obesity itself. In addition, even though previous studies conducted by our group did not document an association between the Traditional-Lebanese pattern and obesity risk [48], the results of this study showed that higher adherence to the Lebanese pattern was associated with higher odds of MHOv/O in overweight and obese adults. Taken together, these findings suggest that, although the Traditional-Lebanese dietary pattern may not be protective against obesity, it may offset or buffer adiposity-related metabolic abnormalities in overweight and obese subjects. The Traditional-Lebanese dietary pattern, a variant of the Mediterranean diet [37], is in fact a pattern that is rich in fruits, vegetables, legumes, olives, olive oil, and dairy products. This dietary pattern was also found to be associated with higher intakes of monounsaturated fats, polyunsaturated fats, fiber, and calcium while being characterized by lower intakes of protein, cholesterol, saturated fat, and sugars [25]. Dietary fiber, through its colonic, intrinsic and hormonal effects, and mono- and polyunsaturated fats, through their ability to buffer lipid and insulin fluctuations and peaks, may work in concert to increase insulin sensitivity, enhance fat oxidation, and decrease cardio-metabolic risk [49,50,51,52]. In addition, the beneficial combinations of phytochemicals, antioxidants, and fiber brought by a diet rich in legumes, fruits, and vegetables may decrease oxidative stress, temper the inflammatory response, and therefore enhance insulin sensitivity [53]. Dairy products, which also characterize the Lebanese dietary pattern, have been associated with decreased visceral fat, an effect that is likely to be mediated by independent or synergistic effects of calcium and dairy protein, on lipolysis, lipogenesis, and thermogenesis [54,55]. Our findings related to the positive association between the Lebanese dietary pattern and the MHOv/O phenotype are similar to those reported by Phillips et al. [20], whereby in a cross-sectional study among adults aged 45–74 years, greater compliance with the food pyramid recommendations and higher dietary quality were positively associated with metabolic health in obese subjects. 

The findings of this study ought to be considered within the context of its limitations. First, we have included in our sample both overweight and obese individuals and not only obese. Descriptive statistics, however, have demonstrated that there was no significant difference in BMI between MHOv/O and MUHOv/O, indicating that overweight individuals did not have higher odds of belonging to the MHOv/O phenotype over obese participants. Second, the MHOv/O state may not be a stable phenotype, and there remain open questions related to whether MHOv/O represents a transient phenotype changing with aging and behavioral and environmental factors [56]. Third, the percentage of survey participants who agreed to give blood (respondents) was low (24.3%). Comparison of respondents and non-respondents showed that both groups were comparable across the sociodemographic characteristics except for marital status and proportions of overweight and obesity (as indicated in the Methods section). Lastly, the cross-sectional design of this study does not allow for causality inference. Longitudinal studies are needed to further confirm the role of dietary patterns in modulating metabolic profiles in high risk individuals. 

## 5. Conclusions 

In conclusion, the study showed that overweight and obese subjects with higher adherence to the Traditional-Lebanese dietary pattern had higher odds of belonging to the MHOv/O phenotype, independent of other socio-demographic, lifestyle characteristics. These findings suggest that the Traditional-Lebanese dietary pattern, a variant of the Mediterranean diet, may be associated with offset or delayed development of adiposity-related metabolic abnormalities. In this context, interventions and strategies aiming at preserving and promoting the traditional diet could be proposed in Lebanon. It is important to note that, even though this study has identified factors that modulate the odds of MHOv/O, maintaining a healthy body weight remains the most impactful public health recommendation to decrease metabolic abnormalities and associated diseases.

## Figures and Tables

**Table 1:** Descriptive characteristics of the study population and their association with Metabolically Healthy Overweight and Obesity (MHOv/O), as derived by simple logistic regression (*n* = 196).

	MUHOv/O (*n* = 123)	MHOv/O (*n* = 73)	*p*-Value ^†^	OR (95% CI)
Age (years)	42.7 ± 15.7	39.2 ± 13.0	0.12	0.98 (0.96–1.00)
Sex			0.00	
Males	81 (66)	29 (40)		Ref
Females	42 (34)	44 (60)		2.93 (1.61–5.33)
Marital status			0.93	
Single	38 (31)	23 (32)		Ref
Married	85 (69)	50 (68)		0.97 (0.52–1.81)
Education			0.06	
Middle school	60 (49)	23 (31)		Ref
High school	32 (26)	27 (37)		1.93 (0.94–3.99)
University & Higher education	31 (25)	23 (32)		2.20 (1.09–4.44)
Family history of obesity			0.84	
No	69 (56)	42 (57)		Ref
Yes	54 (44)	31 (43)		0.94 (0.523–1.69)
Crowding index	1.0 ± 0.5	1.2 ± 0.5	0.10	1.56 (0.91–2.66)
<1person per room	52 (43)	22 (31)		Ref
≥1 person per room	70 (57)	50 (69)		1.69 (0.91–3.13)
Breakfast per week	5.0 ± 2.8	5.3 ± 2.5	0.40	1.05 (0.94–1.17)
Breakfast Skippers (≤5 times/week)	44 (36)	24 (33)		Ref
Breakfast consumers (>5 times/week)	79 (64)	49 (67)		1.14 (0.62–2.10)
Smoking			0.24	
No	83 (67)	55 (75)		Ref
Yes	40 (33)	18 (25)		0.68 (0.35–1.30)
Physical activity level			0.00	
Low	49 (40)	18 (25)		Ref
Moderate	29 (24)	9 (12)		0.85 (0.34–2.13)
High	45 (36)	46 (63)		2.78 (1.41–5.49)
Snack per day	1.5 ± 1.2	1.5 ± 0.9	0.60	0.93 (0.71–1.23)
Eating at TV per week	2.6 ± 3.1	2.4 ± 3.2	0.57	0.97 (0.89–1.07)
Eating out per week	1.5 ± 2.2	0.8 ± 1.4	0.03	0.84 (0.70–1.01)
BMI (Kg/m^2^)	30.2 ± 4.1	29.3 ± 3.7	0.13	0.94 (0.87–1.02)
Waist circumference (cm)	97.4 ± 11.1	92.6 ± 10.4	<0.01	0.95 (0.93–0.98)
Insulin (μU/mL)	29.5 ± 22.6	21.2 ± 8.3	<0.01	0.95 (0.91–0.98)
CRP (mg/dl)	6.9 ± 9.2	5.6 ± 5.6	0.29	0.985 (0.93–1.02)
Percent body fat	32.0 ± 7.6	32.7 ± 6.9	0.56	1.01 (0.97–1.06)

Numbers in this table represent mean ± SD for continuous variables and *n* (%) for categorical variables. ^†^ *p*-values were derived from chi square test for categorical variables and from independent samples *t*-test for continuous variables.

**Table 2:** Multiple logistic regression for the association between socio-demographic and lifestyle characteristics with MHOv/O in the study population (*n* = 196).

Demographic and Lifestyle Variables	OR (95% CI)
Age (years)	0.99 (0.97–1.02)
Sex	
Males	Ref
Females	3.81 (1.95–7.40) **
Education	
Middle school	Ref
High school	2.66 (1.19–5.96) *
University & Higher education	2.49 (1.10–5.70)
Physical activity level	
Low	Ref
Moderate	0.59 (0.22–1.60)
High	2.35 (1.13–4.92) *

* *p*-value ≤ 0.05. ** *p*-value ≤ 0.001.

**Table 3:** Crude and adjusted logistic regression models describing the association between the various dietary patterns and MHOv/O in the study population, as derived by logistic regression (*n* = 196) *^,^^†^.

	Crude Model OR; 95% CI	Adjusted Model OR; 95% CI
**Fast Food/Dessert**		
Low adherence	Ref	Ref
High adherence	0.79 (0.43–1.45)	1.38 (0.66–2.92)
**Traditional-Lebanese Pattern**		
Low adherence	Ref	Ref
High adherence	1.29 (0.69–2.40)	1.83 (1.09–3.91)
**High Protein Pattern**		
Low adherence	Ref	Ref
High adherence	1.17(0.63–2.17)	1.36 (0.69–2.70)

* Low adherence was defined as belonging to the 1st tertile of the pattern score, while high adherence was defined as belonging to the 2nd and 3rd tertiles. ^†^ The adjusted model included sex, education, and physical activity.

## Appendix

**Table A1:** Food items/groups constituting the dietary patterns prevalent in the study population *^,†^.

Dietary Patterns		
Fast Food/Dessert	Traditional-Lebanese	High protein
Hamburger (0.76)	Dairy products-full fat (0.58)	Fish (0.70)
Shawarma (0.72)	Olives (0.56)	Chicken (0.69)
Pizza and pies (0.70)	Fruits and vegetables (0.49)	Meat (0.60)
Falafel Sandwiches (0.61)	Legumes (0.47)	Dairy products-low fat (0.54)
Desserts (0.41)	Grains (0.47)	Breakfast cereals (0.23)
Carbonated beverages and juices (0.4)	Eggs (0.45)	-
Mayonnaise (0.35)	Vegetable oil (0.43)	-
Butter (0.22)	Nuts and dried fruits (0.40)	-
Alcoholic beverages (0.2)	Traditional sweets (0.37)	-

* Factor loading of the various food groups/items are presented in (). ^†^ The dietary patterns and the food items-and their factor loading-making up these patterns were taken from Naja et al. (2013) [29].
